# Discovering the cost of care: consumer, provider, and retailer surveys shed light on the determinants of malaria health-seeking behaviours

**DOI:** 10.1186/s12936-016-1232-7

**Published:** 2016-03-22

**Authors:** Amruta Dixit, Ming-Chieh Lee, Brittany Goettsch, Yaw Afrane, Andrew K. Githeko, Guiyun Yan

**Affiliations:** Program in Public Health, University of California Irvine, Irvine, CA USA; St. Joseph Heritage Healthcare, Anaheim, CA USA; Centre for Global Health Research, Kenya Medical Research Institute, Kisumu, Kenya

## Abstract

**Background:**

The growing threat of insecticide resistance in mosquitoes and drug resistance in the *Plasmodium* parasites increases the importance of ensuring appropriate malaria case management and enabling positive health-seeking behaviour. Treatment-seeking behaviours are poorly characterized in malaria-endemic regions that have been the focus of intensive control and elimination campaigns. This study uses a comprehensive approach to shed light on the determinants of malaria treatment-seeking behaviours from different perspectives.

**Methods:**

The authors conducted cross-sectional surveys from 832 households, fifteen health centers, and 135 retailers across three sites in the Emuhaya and Kakamega districts of the western Kenyan highlands. Participants were recruited via random sampling and data were collected with the use of a structured questionnaire about malaria treatment-seeking behaviour. All households, healthcare facilities, and retailers were mapped using a handheld GPS and a GIS algorithm was used to calculate “walk distance” based on the Tobler rule; an estimate of this distance was used to calculate the travel time used in the analyses.

**Results:**

Across the three sites, 47.5–78.9 % of the residents sought diagnosis and treatment at hospitals, clinics, or dispensaries; 6.3–26.1 % of the residents sought malaria care only at pharmaceutical retailers. Overall, 40.3–59.4 % of residents reported delaying seeking care for more than 24 h after fever onset. After adjustment, residents who chose to visit a pharmaceutical retail facility rather than a hospital were 121 and 307 % more likely to delay seeking medical care after fever onset than those who reported choosing a healthcare facility for treatment. No significant association was found between travel time and delay in seeking care. The surveys of the healthcare facilities indicated an average total cost per patient per visit was 112 KES ($1.40 US) for public facilities and 165 KES ($2.06 US) for private facilities.

**Conclusion:**

Understanding the local health behaviours that perpetuate transmission of malaria will help develop targeted preventive measures and educational interventions that can empower the residents with the knowledge needed to combat malaria in a safe and effective manner. Ensuring patient access to health care facilities in countries with high disease burdens has broader implications on measures of equity and on public health prevention methodologies.

**Electronic supplementary material:**

The online version of this article (doi:10.1186/s12936-016-1232-7) contains supplementary material, which is available to authorized users.

## Background

In spite of intensified malaria control efforts, malaria is a major public health problem, particularly in Africa. Globally, malaria is estimated to affect 200 million people and kill more than 500,000 people per year, mostly children under the age of five [1]. In the past decade, Africa has seen a vast increase in coverage with vector control interventions, with almost half of the susceptible population receiving access to insecticide-treated bed nets [1]. Additionally, indoor residual spraying in Africa protected over 55 million people from malaria [1]. However, with the growing threat of insecticide resistance in mosquitoes [2, 3] and drug resistance in the parasites [4, 5], ensuring appropriate malaria case management and enabling positive health-seeking behaviour grows in importance.

The health behaviours of the local populace are intrinsically linked to case management policies. Sensitive and accurate diagnosis and timely treatment with effective drugs are key components of the World Health Organization (WHO) malaria treatment guidelines [6]. However, without an understanding of the treatment-seeking behaviours of the susceptible population as well as a careful evaluation of the determining factors of those behaviours, malaria control and elimination programmes may be fragmented from the reality seen in the field, and consequently intervention strategies may not be effective or sustainable.

An important variable in the determination of health-seeking behaviour is access to health care services. According to the WHO, people living within 1 h of travel time of a health care facility are generally considered to have access to health care [7]. The inverse relationship between distance to facility and use of health care services has been well established [8–10]. However, it has largely been based on measures of Euclidean distance [7] even when the topography and transport infrastructure in the area rarely allow for a direct path to the facility. In addition to travel impedances, other factors that can affect health-seeking behaviour include affordability and availability of medicines and medical care [11]. Previous studies have shown that in the absence of access to trained medical personnel, people will choose to receive information from untrained sources such as the local chemist or pharmaceutical retailer [12, 13]. Self-medication or home treatment of malaria has generally been shown to have a lower cure rate than treatment in an institutional setting [14] and the tendency to self-diagnose malaria and subsequently self-medicate, has been growing in regions of Africa with limited healthcare access [15, 16]. These various factors all have an impact on the perpetuation of regional malaria transmission.

In Kenya, 76 % of the population is at risk for malaria; in 2013, there were over 2.3 million confirmed cases of malaria in the country [1]. It is crucial to ensure that people are seeking appropriate diagnosis and anti-malarial treatment in a timely manner to reduce malaria mortality, morbidity, and transmission. As treatment-seeking behaviour and healthcare utilization has been shown to be affected by the multitude of factors listed above, we attempted to exact a more comprehensive measure by integrating opinions and habitudes from consumers, health providers, and retailers. The holistic nature of the study is especially appropriate at a time where malaria control efforts such as mass distributions of bed nets and subsidy of artemisinin-based combination therapy (ACT) have intensified, but without a suitable adjustment for health-seeking behaviour patterns of the study population.

The objective of this study was to determine the malaria treatment-seeking behaviour patterns in the western Kenya highlands, and to elucidate some of the major perceived hindrances to healthcare access in that region. The residents’ perceptions regarding barriers may impinge upon their ability to seek diagnoses and treatment at a healthcare facility in a timely manner. This discordance between perceptions and reality may serve as a critical target point in an effective malaria control or elimination programme.

## Ethical considerations

The project was approved by the Institutional Review Board of UC-Irvine and Ethical Review Committee of Kenya Medical Research Institute.

## Methods

### Study area and study population

**Fig. 1:**
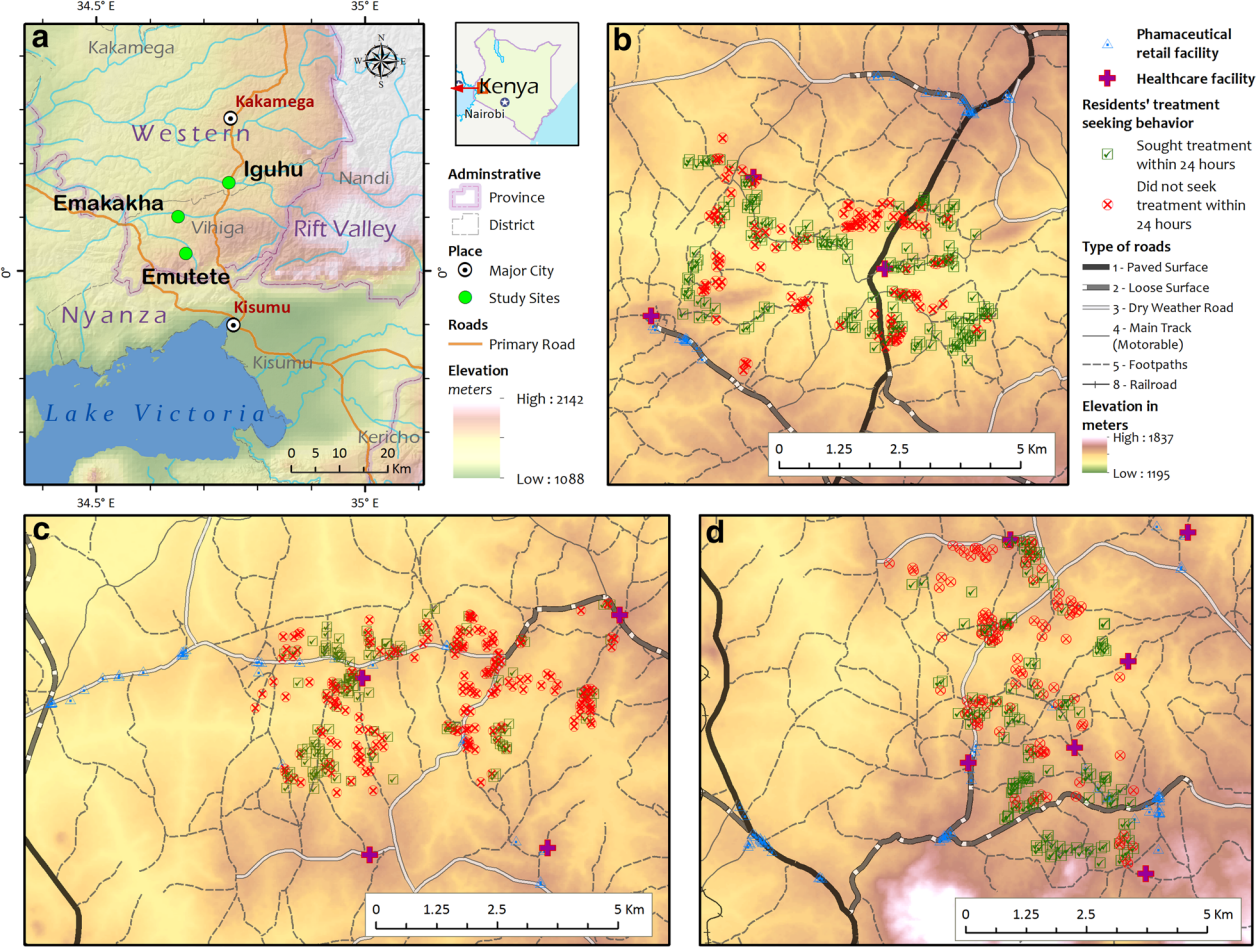
**a** Overview of all study sites. Location of study sites within Kenya. **b, c, d** Iguhu, Emakakha, and Emutete study areas. Each map focuses on the individual study area and shows the distribution of residents’ healthcare seeking patterns. The individual study area’s healthcare facilities and retail outlets are also shown along with its roads and access paths

The authors conducted cross-sectional surveys in three study sites, and data were collected from 832 households, 15 health centres, and 135 retailers in the Emuhaya and Kakamega districts in the western Kenyan highlands (Fig. 1a). The study sites included three sub-locations: Iguhu (34˚44′E, 0˚11′N, 1430–1580 m above sea level) in Kakamega district (Fig. 1b); and Emakakha (34˚39′E, 0˚07′N, 1460–1520 m above sea level) (Fig. 1c) and Emutete (34˚38′E, 0˚02′N, 1480–1640 m above sea level) (Fig. 1d) in Emuhaya district. Each site was 3 × 6 km^2^ and each was composed of several villages. These sites were used for other vector ecology and malaria epidemiology research by other members of the research team [17–19]. The topography of the study area consists of hills, valleys, and plateaus and a variety of land use and land cover patterns exist. This region generally experiences two rainy seasons (April–May and October–November) and two dry seasons (January–February and July–August) [13] although during the year the survey was conducted, the rainy season started later than in previous years and lasted well into August 2011 [6, 14].

### Local resident survey

In each of the three study sites, the questionnaire was administered to an adult member of the randomly selected households. Informed consent was obtained from every participant in the study. All households were mapped using handheld global positioning systems (GPS) (Garmin). The predominant tribe in the area is the Luhya tribe and thus, all surveys were translated from English and orally administered in the Luhya language. Survey questions were used to acquire demographic and socioeconomic information and to determine the participants’ treatment-seeking behaviour with regards to malaria. The survey also included questions pertaining to treatment facility choices and treatment affordability. All options were read out to the respondents before their answer was recorded. The surveys were conducted by local, trained technicians, over a period of several months, beginning in mid-July and ending in mid-December.

### Health facility survey

To assess coverage and utilization of health facilities by local residents, all healthcare facilities within the three study areas were mapped and surveyed by field staff. The initial step was to acquire the complete list of health facilities from KEMRI (Kenya Medical Research Institute). The list contained records of more than 100 facilities in the Western Province with information on services offered, the approximate location, and the second to fifth administrative level in which each facility was located. Second, each health facility on the topographic map from Kenya Geological Survey was located and health facilities that fell within a ten kilometer radius of the study area and were thus, most likely to serve potential study participants were selected; there were fifteen health facilities on the final list. The team visited each health facility on the final list, recorded the facility’s GPS coordinates, and administered the questionnaire to the medical staff member in charge. The information acquired included hours and days of operation, the number and type of medical staff, malaria diagnosis method, any facility-imposed charges associated with a suspected malaria visit, and the storage and supply of anti-malarials.

### Pharmaceutical retail facility survey

Given that a considerable proportion of residents purchase anti-malarials from pharmaceutical retail facilities, it is necessary to include such facilities in the surveillance. The team searched for and interviewed all potential outlets along roads and markets where retail facilities are normally located. Pharmaceutical retail facility owners were asked to respond to questions regarding the presence of a licensed pharmacist on site, approximate number of customers served per day, types of anti-malarials in stock, cost for anti-malarials and their supplier for the medication. A total of 135 retailers were surveyed. A small, random sample of these outlets (eight in total) was chosen and one prescription of artemether-lumefantrine (AL, Coartem^®^) was purchased from each of the chosen outlets in the sample to test for the levels and presence of the pharmacologically relevant active compound by means of ELISA and HPLC [20, 21]. The tests were performed according to the protocols described by Wang et al. [21].

### Data analysis

A total of 832 households, 15 healthcare facilities, and 135 pharmaceutical retail facilities across three study sites participated in the study. All survey responses were entered into MS Excel and the coordinates from the GPS readings were transferred to ArcGIS 10.2 to generate the necessary maps (Fig. 1). A GIS algorithm to calculate “walk distance” was created based on the Tobler rule [22]. The estimate of this distance was used to calculate the travel time used in the remaining analyses. For more information, please refer to Additional file 1.

One of the major purposes of the survey was to determine whether or not the participants sought diagnosis and treatment within 24 h of fever onset. Logistic regression was performed on the individual survey data using the likelihood of delay as the binary outcome variable. The outcome of a delay in seeking diagnosis and treatment was regressed on key sociodemographic and health-seeking behaviour variables, both individually and in combination. The results of the bivariate analyses and an adjusted multivariate model are included below. When adjusting the multivariate model, both, variables that have been determined as important in the literature as well as variables that measured the participants’ perceived barriers were included in the model. Thus, socioeconomic and demographic variables were necessarily included regardless of their significance in the bivariate analyses. The other included variables served as indicators of accessibility, availability of drugs, and affordability.

The missing data were imputed 100 times using the predictive mean matching method using the “mice” package in R 3.1.3. All the regression analyses presented below were performed on the imputed datasets separated by site, rather than as a complete case analysis. Statistical analyses were conducted in R 3.1.3 and MS Excel 2010.

## Results

### Demographic and socioeconomic characteristics

Among all sites, the majority of households were headed by men (Table 1). Overall, 63 % of household heads had only a primary school education or less. At least 91 % of the participants across all three sites live in mud homes and approximately 85 % of residents in all sites own both furniture and livestock.

**Table 1:** Socioeconomic and demographic characteristics of study participants in western Kenya highlands

Variable	Emakakha (N = 303)	Emutete (N = 256)	Iguhu (N = 273)	Total (N = 832)
Demographic variables				
Primary income earner education				
Did not finish primary	42 (13.9 %)	62 (24.2 %)	75 (27.5 %)	179 (21.5 %)
Primary school	137 (45.2 %)	114 (44.5 %)	97 (35.5 %)	348 (41.8 %)
Secondary school and beyond	123 (40.6 %)	79 (30.9 %)	101 (37.0 %)	303 (36.4 %)
Missing	1 (0.3 %)	1 (0.4 %)	0	2 (0.2 %)
Sex of primary income earner				
Male	236 (77.89 %)	144 (55.9 %)	196 (71.8 %)	576 (69.2 %)
Female	66 (21.8 %)	107 (41.8 %)	75 (27.5 %)	248 (29.8 %)
Both sexes	0	2 (0.8 %)	2 (0.7 %)	4 (0.5 %)
Missing	1 (0.3 %)	5 (3.5 %)	0	6 (0.7 %)
Socioeconomic indicators				
Possessions				
Ownership of both furniture and livestock	268 (88.4 %)	238 (93.0 %)	204 (74.7 %)	710 (85.3 %)
Ownership of furniture only	29 (9.5 %)	15 (5.8 %)	48 (17.5 %)	92 (11.1 %)
Ownership of livestock only	2 (0.7 %)	3 (1.2 %)	19 (7.0 %)	24 (2.9 %)
None	2 (0.7 %)	0	0	2 (0.2 %)
Missing	2 (0.7 %)	0	2 (0.8 %)	4 (0.5 %)
Home construction				
Mud	276 (91.0 %)	235 (91.8 %)	253 (92.7 %)	764 (91.8 %)
Cement/brick	22 (7.3 %)	17 (6.6 %)	17 (6.2 %)	56 (6.7 %)
Missing	5 (1.7 %)	4 (1.6 %)	3 (1.1 %)	12(1.4 %)
Malaria status				
No malaria infection in family in past year	3 (1.0 %)	0	4 (1.5 %)	7 (0.8 %)
At least one victim in family in past year	291 (96.0 %)	205 (80.1 %)	242(88.6 %)	738 (88.7 %)
Missing	9 (3.0 %)	51 (19.9 %)	27 (9.9 %)	87 (10.5 %)
Health-seeking behaviours				
Action taken upon suspicion of malaria				
Treatment sought at hospitals, clinics, and dispensaries exclusively	144 (47.5 %)	202 (78.9 %)	204 (74.7 %)	550 (66.1 %)
Treatment sought at pharmaceutical retail facilities exclusively	79 (26.1 %)	16 (6.3 %)	25 (9.2 %)	120 (14.4 %)
Treatment sought at traditional healers exclusively	1 (0.3 %)	0	0	1 (0.1 %)
No treatment facility preference indicated	42 (13.9 %)	9 (3.5 %)	25 (9.2 %)	76 (9.1 %)
No action taken	37 (12.2 %)	24 (9.4 %)	18 (6.6 %)	79 (9.5 %)
Missing	0	5 (1.9 %)	1 (0.3 %)	6 (0.7 %)
Treatment seeking timeline				
Delay treatment for >24 h after fever onset	180 (59.4 %)	117 (45.7 %)	110 (40.3 %)	407 (48.9 %)
Seek treatment within 24 h after fever onset	119 (39.3 %)	135 (52.7 %)	160 (58.6 %)	414 (49.8 %)
Missing	4 (1.3 %)	4 (1.6 %)	3 (1.1 %)	11 (1.3 %)
Medicine				
Artemisinin combination therapy (ACT) exclusively	172 (56.8 %)	149 (58.2 %)	147 (53.9 %)	468 (56.3 %)
Non-ACT exclusively: quinine, SP, Fansidar	46 (15.2 %)	16 (6.3 %)	25 (9.2 %)	87 (10.5 %)
Painkillers exclusively	10 (3.3 %)	6 (2.3 %)	2 (0.7 %)	18 (2.2 %)
Combination of ACTs, non-ACT, and painkillers	71 (23.4 %)	80 (31.3 %)	94 (34.4 %)	245 (29.4 %)
Missing	4 (1.3 %)	5 (1.9 %)	5 (1.8 %)	14 (1.7 %)
Facility where pharmaceutical treatment is purchased				
Hospitals, clinics, and dispensaries exclusively	153 (50.5 %)	188 (73.4 %)	174 (63.7 %)	515 (61.9 %)
Shopkeepers and chemists exclusively	110 (36.3 %)	24 (9.4 %)	56 (20.5 %)	190 (22.8 %)
No preference indicated	37 (12.2 %)	41 (16.0 %)	43 (15.8 %)	121 (14.5 %)
Missing	3 (1.0 %)	3 (1.2 %)	0	6 (0.7 %)
Health access measures				
Self-reported nearest facility				
Hospitals, clinics, and dispensaries exclusively	180 (59.4 %)	227 (88.7 %)	243 (89.0 %)	650 (78.1 %)
Shopkeepers and chemists exclusively	109 (36.0 %)	16 (6.2 %)	25 (9.1 %)	150 (18.0 %)
Comparable distance to hospitals/clinics/dispensaries and pharmaceutical retail facilities	12 (3.9 %)	12 (4.7 %)	4 (1.5 %)	28 (3.4 %)
Missing	2 (0.7 %)	1 (0.4 %)	1 (0.4 %)	4 (0.5 %)
GIS-calculated nearest facility				
Health care center exclusively	93 (30.7 %)	158 (61.7 %)	248 (90.8 %)	499 (60.0 %)
Pharmaceutical retailer exclusively	209 (69.0 %)	95 (37.1 %)	17 (6.2 %)	321 (38.6 %)
Equidistant	1 (0.3 %)	3 (1.2 %)	8 (3.0 %)	12 (1.4 %)
Travel time				
Mean self-reported travel time to nearest facility	54.9 min (95.4 %)	55.1 min (98.0 %)	47.1 min (96.0 %)	52.4 min (96.5 %)
Missing	14 (4.6 %)	5 (2.0 %)	11 (4.0 %)	30 (3.6 %)
Average GIS-calculated travel time				
Health care center	32.5 min	25.5 min	27.6 min	85.6 (28.5 %)
Retailer	21.9 min	27.0 min	57.6 min	106.5 (35.5 %)
Method of travel				
Walk exclusively	260 (85.8 %)	200 (78.1 %)	240 (87.9 %)	700 (84.1 %)
Bicycle	30 (9.9 %)	16 (6.2 %)	10 (3.7 %)	56 (6.7 %)
Car or other motorized transport	8 (2.6 %)	25 (9.8 %)	10 (3.7 %)	43 (5.2 %)
Mix of walking, biking, and motorized transport	3 (1.0 %)	12 (4.7 %)	11 (4.0 %)	26 (3.1 %)
Missing	2 (0.7 %)	3 (1.2 %)	2 (0.7 %)	7 (0.8 %)
Reason for delay (if any)				
Do not delay	2 (0.7 %)	0	1 (0.4 %)	3 (0.4 %)
Money	182 (60.0 %)	98 (38.3 %)	89 (32.6 %)	369 (44.4 %)
Distance	2 (0.7 %)	2 (0.8 %)	1 (0.4 %)	5 (0.6 %)
Expect improvement of condition	46 (15.2 %)	50 (19.5 %)	33 (12.1 %)	129 (15.5 %)
Transportation	1 (0.3 %)	0	1 (0.4 %)	2 (0.2 %)
Mix	15 (4.9 %)	1 (0.4 %)	5 (2.1 %)	21 (2.5 %)
Other	0	0	1 (0.4 %)	1 (0.1 %)
Missing	55 (18.2 %)	105 (41.0 %)	142 (52.0 %)	302 (36.3 %)
Affordability of treatment				
Found treatment unaffordable	43 (14.2 %)	85 (33.2 %)	99 (36.3 %)	227 (27.3 %)
Found treatment affordable	245 (80.8 %)	169 (66.0 %)	174 (63.7 %)	588 (70.7 %)
Missing	15 (5.0 %)	2 (7.8 %)	0	17 (2.0 %)

### Health-seeking behaviours

Although at least 80 % of all households surveyed had experienced a malaria infection in the family in the past year, about 10 % of the participants indicated that they take no action upon malaria symptom onset (Table 1). In Iguhu and Emutete, approximately 77 % of the residents sought diagnosis and treatment at hospitals, clinics, or dispensaries; fewer than 10 % of the residents sought malaria care only at pharmaceutical retailers. However, in Emakakha, fewer than 48 % of residents sought diagnosis and treatment at a hospital or a clinic and more than 26 % of them chose pharmaceutical retailers as their first choice of treatment facility. Overall, a sizeable proportion of residents (40.3–59.4 %) reported delaying seeking care more than 24 h after fever onset. The most common reason for the delay was a lack of funds, followed by an expectation of improvement in condition (Table 1).

In spite of long-standing WHO guidelines recommending ACT as the first-line treatment for malaria, only 56 % of all participants indicated that they used ACT to treat malaria. More than 29 % of all respondents indicated multiple drugs, including quinine, chloroquine, and sulfadoxine-pyrimethamine (SP), as possible options for malaria treatment. This suggests an overall propensity for choosing treatment based on availability of medication rather than on highest degree of efficacy.

### Health access measures

Most participants (84 % overall) chose to walk to the treatment facility of their choice (Table 1). All participants were found to be living less than 1 h of travel time from a hospital or clinic (maximum calculated time across all sites: 50 min) based on our calculations in ArcGIS^®^. However, patients significantly overestimated the amount of time it would take to walk to the nearest facility (paired *t* test, p < 0.001). For those who reported that the nearest treatment facility was a hospital or clinic, the self-reported travel time to the hospital was overestimated by approximately 22 min in Emutete and Iguhu and by over 28 min in Emakakha. For those who indicated that the nearest facility was a pharmaceutical retailer, the overestimation of travel time varied by site (18 min in Iguhu, 40 min in Emutete, and 33 min in Emakakha). Of those who sought care exclusively from pharmaceutical retail facilities, 14 % did so despite being further away from these facilities than from the nearest hospital or clinic.

### Risk factor analysis for delay in seeking care

Overwhelmingly, participants who chose to delay seeking medical care for more than 24 h after fever onset were more likely to visit the pharmaceutical retailer to purchase medication rather than visit the hospital to seek diagnostic workup and treatment. This association was significant and strongly pronounced across all three sites (Table 2). It was reflected in the bivariate analyses and stayed significant even after adjustment for other variables. When all other potentially influential variables were accounted for, residents who chose to visit a pharmaceutical retail facility rather than a hospital were more likely to delay seeking medical care after the onset of malaria by between 121 and 307 % than those who reported choosing a healthcare facility for malaria treatment (Table 3).

**Table 2:** Bivariate analyses of relevant risk factors for odds of delaying treatment ≥ 24 h by study site

Variable	Emakakha OR [95 % CI]	Emutete OR [95 % CI]	Iguhu OR [95 % CI]
Lived in mud home	*4.21 [1.60, 11.04]*	3.02 [0.96, 9.52]	3.42 [0.95, 12.2]
Ref: lives in cement/brick home	1	1	1
Owned either furniture or livestock or neither	0.77 [0.37, 1.60]	0.81 [0.30, 2.21]	0.79 [0.45, 1.41]
Ref: owns both furniture and livestock	1	1	1
Wage head has a primary school education	0.98 [0.59, 1.63]	*1.89 [1.04, 3.42]*	1.03 [0.58, 1.83]
Wage head did not finish primary school	0.63 [0.31, 1.27]	1.65 [0.83, 3.26]	1.45 [0.79, 2.67]
Ref: wage head finished secondary school or beyond	1	1	1
Female wage head of household	1.34 [0.76, 2.36]	1.63 [0.98, 2.72]	*3.41 [1.96, 5.94]*
Ref: male wage head of household	1	1	1
Chose pharmaceutical retailers for treatment	*3.86 [2.36, 6.30]*	*2.79 [1.42, 5.50]*	*1.86 [1.06, 3.28]*
Ref: chose healthcare facility for treatment	1	1	1
Self-reported nearest facility was a pharmaceutical retailer	*2.12 [1.30, 3.45]*	*5.05 [1.97, 12.93]*	*0.20 [0.07, 0.59]*
Ref: self-reported nearest facility was a healthcare facility	1	1	1
Walked to facility when seeking treatment	1.11 [0.57, 2.18]	0.59 [0.32, 1.08]	2.24 [0.96, 5.21]
Ref: took a car or other motorized transport to facility when seeking treatment	1	1	1
Found treatment to be unaffordable	*2.64 [1.31, 5.31]*	1.28 [0.76, 2.16]	*0.56 [0.33, 0.95]*
Did not find treatment to be unaffordable	1	1	1

*SSgnificant at α <0.0*

*OR* odds ratio, *CI* confidence interval

**Table 3:** Multivariate (adjusted) model of relevant risk factors for odds of delaying treatment ≥24 h by study site

Variables	Emakakha OR [95 % CI]	Emutete OR [95 % CI]	Iguhu OR [95 % CI]
Lived in mud home	*8.32 [2.58, 26.90]*	2.75 [0.78, 9.74]	2.39 [0.60, 9.61]
Ref: lives in cement/brick home	1	1	1
Owned either furniture or livestock or neither	0.98 [0.42, 2.27]	0.88 [0.31, 2.55]	0.65 [0.34, 1.26]
Ref: owns both furniture and livestock	1	1	1
Wage head has a primary school education	0.82 [0.46, 1.47]	1.60 [0.82, 3.14]	0.94 [0.49, 1.79]
Wage head did not finish primary school	0.45 [0.20, 1.03]	1.46 [0.67, 3.21]	0.99 [0.49, 1.99]
Ref: wage head finished secondary school or beyond	1	1	1
Female wage head of household	1.29 [0.68, 2.45]	1.69 [0.96, 3.0]	*3.13 [1.72, 5.69]*
Ref: male wage head of household	1	1	1
Chose pharmaceutical retailers for treatment	*4.07 [2.31,7.21]*	*2.21 [1.07, 4.61]*	*2.60 [1.35, 4.99]*
Ref: Chose healthcare facility for treatment	1	1	1
Self-reported nearest treatment facility is pharmaceutical retailer	1.37 [0.76, 2.49]	*4.23 [1.55, 11.53]*	*0.15 [0.04, 0.48]*
Ref: self-reported nearest facility was a healthcare facility	1	1	1
Walked to facility when seeking treatment	1.10 [0.5, 2.42]	*0.48 [0.24, 0.95]*	1.91 [0.76, 4.81]
Ref: took a car or other motorized transport to facility when seeking treatment	1	1	1
Found treatment to be unaffordable	*5.36 [2.24, 12.81*]	1.15 [0.64, 2.06]	0.59 [0.33, 1.06]
Did not find treatment to be unaffordable	1	1	1

*Significant at α <0.05*

*OR* odds ratio, *CI* confidence interval

### Healthcare facility characteristics

There were fifteen healthcare facilities that served the study population. Of the 15, 11 were publicly administered by the Kenya Ministry of Health and four were privately owned and operated (Table 4). It was found that 40 % of them exhausted their stores of artemisinin-based malaria treatment at least one or more times per month; six out of the 15 encountered a shortage more frequently. Furthermore, the average cost to patients per visit for malaria treatment at a public facility was 72 KES (equivalent to $0.90 US at the time) and 125 KES ($1.56 US) at a private facility. The costs were reflective of the admission or registration fees, diagnosis fees, and other miscellaneous charges. These charges did not include the cost of the subsidized ACT drugs (40 KES, $0.50 US, to be paid for by the patient) bringing the average total cost per patient per visit up to 112 KES ($1.40 US) for public facilities that did not have ACT in stock and 165 KES ($2.06 US) for private facilities.

**Table 4:** Summary of health care facility (hospitals, clinics, and dispensaries) surveyed

Variable	Public	Private
Number of facilities surveyed	11 (73.3 %)	4 (26.7 %)
# of hospitals	3 (20.0 %)	1 (6.7 %)
# of health centres	5 (33.3 %)	2 (13.3 %)
# of dispensaries/clinics	3 (20.0 %)	1 (6.7 %)
Hours of operation		
9 h or fewer	7 (46.6 %)	4 (26.7 %)
24 h	4 (26.7 %)	0
Days of operation		
5 days/week	5 (33.3 %)	1 (7.0 %)
7 days/week	6 (40.0 %)	3 (20.0 %)
Median population served [range]	13,885 [1310–164,951]	11,189 [2683–20,000]
Staffing		
# of facilities with doctors [range]	2 (13.3 %) [0–8]	1 (7.0 %) [0–3]
Median number of clinical officers [range]	2 [0–18]	1.5 [0–4]
Median number of nurses [range]	8 [2–53]	7 [2–20]
Median number of microscopists [range]	2 [0–4]	2 [2–4]
Malaria		
Median # of microscopy confirmed cases in three mos. preceding survey [range]	222	189
Diagnostic method: microscopy exclusively	5 (33.3 %)	2 (13.3 %)
Diagnostic method: microscopy + RDT	3 (20.0 %)	2 (13.3 %)
Diagnostic method: symptoms exclusively	3 (20.0 %)	0
ACT stocking		
Facilities stocked with ACT at time of survey	11 (73.3 %)	4 (26.7 %)
Experienced shortage of ACT in 3 months preceding survey [range]	4 (26.7 %)	2 (13.3 %)
# of facilities that had to wait >24 h before ACT was restocked	4 (26.7 %)	2 (13.3 %)
# of facilities that either substitute another anti-malarial or refer patient to nearest retailer	5 (33.3 %)	2 (13.3 %)
Charges [in Kenyan shillings]		
# of facilities that charged registration fees [average fee]	11 (73.3 %) [19 KSH]	3 (20.0 %) [55 KSH]
# of facilities that charged diagnosis fees [average fee]	8 (53.3 %) [46 KSH]	4 (26.6 %) [70 KSH]
# of facilities that charged other miscellaneous fees per patient per visit [average fee]	8 (53.3 %) [26 KSH]	0
# of facilities that charged for medication [average fee]	1 (6.7 %) [40 KSH]	3 (20.0 %) [73 KSH]
Overall average costs per patient of health care facility visit (not including cost of medication) [range]	72 KSH [30 KSH–90 KSH]	125 KSH [50 KSH–250 KSH]

Most of the facilities (12) used microscopy in combination with other diagnosing methods to determine infection status as recommended by the WHO; however, three of the health facilities treated patients based on clinical presentation only. Of the 15 clinics and hospitals, 11 (ten public and one private) received their drug supply directly from Kenya Medical Supplies Authority, the medical logistics provider for all Ministry of Medical Services/Public Health supported healthcare facilities in the country. Two private facilities purchased directly from the manufacturer and two others (one private and one public) purchased their ACT stock from local retailers.

### Pharmaceutical retail facility characteristics

Of the 135 pharmaceutical retail facilities interviewed for the study, only nineteen were operated by a licensed chemist or pharmacist. The remaining facilities were operated by small business owners who ran shops in the local markets or along the roadsides. All 19 licensed chemists or pharmacists had anti-malarials available for sale in their facility on the day of the interview. However, only 49 of the 116 non-licensed retailers were found to sell any kind of anti-malarial therapy. Of those 49, only 16 reported the presence of ACT in stock. The remainder sold SP drugs, amodiaquine, quinine, and pain medications. The most commonly stocked antimalarial among the non-licensed retailers were SP drugs; twenty-two shops carried a variety of SP brands. Nine sold amodiaquine and five stocked quinine, either individually or in combination with other therapies. Of all 135 retailers, 38 % served 100 or more customers per day.

The surveys of retailers also served as an opportunity to test for the presence of counterfeit or substandard artemisinin-based anti-malarial drugs in the study sites. Survey staff purchased and tested samples of AL/Coartem^®^ from a random selection of eight retailers and pharmacies associated with healthcare facilities and further testing found that all samples contained the manufacture labelled amount of the artemisinin-derived active compound (Additional file 2), suggesting the quality of ACT being sold met the standard requirements.

## Discussion

Timely and appropriate case management of malaria is integral to the reduction of disease-associated morbidity and mortality. An improvement in the understanding of treatment-seeking behaviours in the susceptible populations can enable the development of targeted interventions that are designed in a manner that is feasible and sustainable within individual communities. However, in spite of intensive interventions in the area, the determinants of treatment-seeking behaviours in our study population have been poorly described.

The results of the current study concur with existing literature to show that medical facilities are largely the primary source of malaria care after fever onset [13]. However, the pharmaceutical retailers are a dominant player in the system and need to be considered as an important variable in any future interventions. Furthermore, the subsidies for ACT provided by the government may be masking the high cost of care imposed by medical facilities, thus driving patients towards cheaper alternatives or to delay seeking care altogether.

The results show a strong association between treatment-seeking delay and choosing to seek treatment at a pharmaceutical retailer rather than visiting a health care facility. This association is significant and present in all sites and holds even after adjustment for other variables. This may be reflective of a reduced perception of severity of malaria that has been shown to occur in areas of medium to high malaria endemicity [23]. Combined with other barriers such as availability of drugs and costs associated with a hospital visit, the low level of perceived severity may be a strong contributor to delaying care.

It was expected that travel time would be a strong barrier of access to medical care in the study population. However, among the participants who responded to the survey regarding their reasons for delaying treatment, a lack of funds stood out as the primary response. Yet, the regression analyses did not show a clear association between delaying treatment and self-reported affordability across the three sites. This may be due to a discrepancy in the participants’ interpretation of affordability based on our survey and the intention of the survey question. Participants may have delayed seeking care at the time of malaria symptom onset because they did not have sufficient funds. However, at the time when they chose to seek treatment, they may have procured the necessary funds and thus, their perception of affordability would have shifted.

Furthermore, the travel time derived for this study, though an improvement upon measures using Euclidean distance, cannot account for the variety of factors that may influence a person’s estimation of the travel time from their home to the nearest healthcare facility. These factors may include road conditions (which vary with the seasons), the ability to gather sufficient funds, preparing oneself or child for travel, and procuring transportation. These estimates, along with other self-reported variables were also susceptible to recall bias, as is the case with many survey-based studies. However, a diligent attempt was made to glean a representative sample of the area by using an appropriate sample size in each site and by randomizing the selection of participants from within the pre-defined areas.

Since surveys of the healthcare facilities indicated that the average cost of a malaria-related visit is between 112 and 165 KES (including the cost of medication), it is clear that is there is discordance in the original intention of the subsidization policy and its implementation. While ACT at government-run facilities was meant to be provided for free to the patient, the frequent stock-outs led the facilities surveyed to refer patients elsewhere, including to private retailers, where the patients may face a greater likelihood of receiving a less effective anti-malarial. Private retailers are not always bound by the subsidization policy and charge for ACT at a higher rate than 40 KES [24]. The ubiquity of various anti-malarial drugs in the market combined with the high charges associated with a hospital or clinic visit may also serve to reduce a patient’s perceived need for a full diagnostic workup.

Accessibility is an important determinant of treatment-seeking behaviour and has implications for the continued transmission of malaria. Some of the variation in health-seeking behaviours between the three study sites may be attributed to the lack of paved roads in the area and the hilly terrain, neither of which are conducive to motor access (see Fig. 1). Most of the participants did not live along major roads. The perceived benefits of receiving a proper malaria diagnosis from a healthcare facility may not be sufficient to outweigh the perceived cost of travel, in terms of both time and effort. Finally, there is a stark difference between the low number of healthcare facilities located in Emakakha and how many more are located in the other two study sites; there is one dispensary and one health centre located within the boundaries of Emakakha and another health centre that lies between the boundaries of Emakakha and Emutete. However, there are several retailers lining the major road that bisects the Emakakha study area, which may help explain why more than one-third of the residents in the site reported a retailer as the nearest treatment facility.

As was observed in the study by Sumba et al., the participants’ decision to seek treatment at a healthcare facility within 24 h of fever onset was not significantly correlated with their socioeconomic status, education level, or proximity to the facility [13]. The strongest determining factor of delay was the decision to choose to seek treatment at a pharmaceutical retailer rather than a medical facility. As such, the observed propensity of the pharmaceutical retailers as seen in other studies [11, 25] to sell medication without appropriate anti-malarial properties has serious negative implications for malaria control and the potential for the spread of artemisinin resistance. A previous experience of being referred to a pharmaceutical retailer when the healthcare facility had depleted its ACT stock may also deter patients from making future visits to the healthcare facility; they may choose to go directly to the retailer, seeing it as a cheaper, faster, and quicker alternative. Future interventions must recognize and include retailers as key players in any control or elimination programme that is to be implemented.

## Conclusion

The year 2010 was the first year that the Affordable Medicines Facility-malaria program to subsidize ACT at private retailers was implemented with the intention of reducing the cost of ACT to 40 KES (equivalent to $0.50 at the time) around the country [26, 27]. However, the discrepancy between the cost of the drug and the full cost of a hospital visit for malaria care was large and posed a significant burden to the affected population. A consideration of change in policy may be needed if the impact is to be significant. These changes may include the implementation of a flat fee for all malaria diagnoses and treatments at public hospitals or an increase in the subsidy of the drug to offset the costs of seeking care at a medical facility. The high cost of seeking diagnosis and treatment at a healthcare facility with appropriate diagnosis may be inhibiting positive health-seeking behaviour and may have a significant impact on the progress of malaria elimination in the area, inadvertently fueling the development and spread of drug resistant parasite strains.

Another point of concern is the relationship between the local health facilities and the pharmaceutical retail outlets. Some facilities not only refer their patients to nearby retailers when ACT is not available in the facility but also indicated that they occasionally receive their supply of ACT from these retailers. Unearthing such relationships can help reveal weak points in control and elimination programmes and allow for the development of directed improvement measures.

Understanding the local health behaviours that perpetuate transmission of malaria will help develop targeted preventive measures, as well as to develop educational interventions that can empower the residents with the knowledge needed to combat malaria in a safe and effective manner. Ensuring patient access to health care facilities in countries with high disease burdens has broader implications on measures of equity and on public health prevention methodologies. It is hoped that the results of this study begin to fill in the gaps in knowledge that would be required to implement policy changes and improve access to health care facilities for the people of the western Kenyan highlands and others in areas experiencing similar malaria transmission patterns.

## Additional files

10.1186/s12936-016-1232-7 Summary of the estimated average travelling speed and cost-weighted factor for potential terrain conditions in western Kenya.
10.1186/s12936-016-1232-7 Comparison between values measured by ELISA and HPLC in the commercial artemisinin-based drugs. The labelled value of active ingredients (a.i.) was all 2.0.
