# Supplementary material for: Discovering the cost of care: consumer, provider, and retailer surveys shed light on the determinants of malaria health-seeking behaviours
Source: Malar J. 2016 Mar 22;15:179. doi: 10.1186/s12936-016-1232-7 (PMC4802645; doi:10.1186/s12936-016-1232-7)
Supplement: Supplementary file 1 — 10.1186/s12936-016-1232-7 Summary of the estimated average travelling speed and cost-weighted factor for potential terrain conditions in western Kenya. [file 12936_2016_1232_MOESM1_ESM.docx]

Supplement 1

Our surveys of area hospitals and clinics, local chemists, and neighborhood retail outlets were designed to shed light on the availability and supply of recommended anti-malarials in our study area of interest. Our field surveys of the residents of the area provided insight into local health-seeking behaviors. In order to best describe and evaluate our measures of accessibility, we employed certain GIS-based methodologies in our analyses. We collected and digitized the detailed spatial information on the population, health facilities, pharmacies, road network, and topographic features influencing the access-time. These were used to develop a geospatial accessibility model based on Tobler’s (1993) equation to estimate travel speeds and subsequent access-time to the nearest health facility and pharmaceutical retailer. The model took into account different topography and transport types and was calibrated using data from actual field survey and observation made by patients seeking treatment.

We assembled all the geo-referenced data, including base map, health facilities, pharmacy, transport network (roads, tracks and barriers), and other topographic features into a geodatabase. We then established our geospatial accessibility model by implementing Tobler’s (1993) equation with the “cost-weighted distance” algorithm within a geographical information system (GIS), ArcGIS Desktop 10.1 (ESRI Inc), to estimate access-time from every 30 x 30 meters grid square to the nearest health facility or pharmaceutical retailer. Separate models were developed for mobilized versus non-mobilized forms of transport and for scenarios with and without access barriers, such as rivers; the likely proportion of residents using each transport type to access facilities in different locations was also estimated. To maximize the realistic estimation in our models, we carried out several calibrations in which survey data on actual access-time reported by survey cases were used to find the optimum model parameters in either mobilized versus non-mobilized forms of transport (Table 3). By combining our estimations and topographic features, we generated several high resolution surfaces under several practical transportation or destination scenarios by 10 minutes access-time intervals.

Walk distance is based on the “Path Distance” tool found in the software. It calculates, for each cell, the least accumulative cost distance to the nearest source, while accounting for surface distance and horizontal and vertical cost factors and weights the speed deduction with the slope change.

$$w=6\times EXP\left( -3.5\times\left| S+0.05 \right| \right)$$

$$w=6\times EXP\left( -3.5\times\left| \tan\frac{Slp}{57.29578}+0.05 \right| \right)$$

-- Tobler (1993)

Here, w is the walking velocity, S is dH/dX = tan (θ), the dimensionless slope; Slp is the degree of slope; the value 57.29578 shown here is a truncated version of the result from 180/π. The unit of velocity w is given in km/hr. This algorithm was created to better estimate pedestrian travelling times. To estimate the travel speeds for crossing different flat terrain, we assumed adults in our study communities could walk at a speed of 5 km/h on field foot path; for off-path travel, we multiplied by a factor of 0.6 to get a walking speed of 3 km/h; for specific land-cover impediments, we used 2.5 km/h to estimate people walking through forested areas and assumed they could maintain a speed of 1 km/h to cross a swampy area. We also assumed people could not cross rivers or water bodies unless there was a bridge.

The traveling speeds for people with vehicle (mainly by car, minibus, or motorcycle) are estimated to be 50 km/h on primary roads (tarmac roads), 30 km/h on secondary roads (gravel roads), and 20 km/h on dry weather road (dirt roads). When traveling on the small, narrow track with bicycle or tricycle, 10 km/h is held as the maximum speed^17^. This was applied to our data from households, retailers, and hospitals and walking times were calculated based on terrain maps used by others within the project. The application of this particular rule is prudent in this case given that a large majority of our participants in all surveyed areas used walking as their primary mode of transportation to reach the health facility (Table 2).

Based on the estimation of travel speeds for each terrain condition, we calculated the cost-weighted factors. The cost-weighted factor represents the potential impedance or the “resistance” in the distance calculation while simulating the difficulty of passing through a specific terrain condition. The higher the value of cost-weighted factor, the higher the impedance of movement on the ground, and the greater the amount of time that is needed to travel an equivalent distance as compared to a cell with lower impedance. Low cost-weighted factors (impedance values) could be assigned to high-speed terrain conditions such as bound surface road, with much larger values for loose surface road or rough terrain. Barriers such as a wide river or a big water body were designated as an inaccessible area, and swamps or forests could be assigned very large impedance values. For instance, if the cost-weighted factor for traveling on a primary road (bound surface) at the speed of 50 km/hr is defined as 1, the factor for traveling on dry weather road (dirt surface- which has lower traveling speeds of 20 km/hr), can be defined at 2.5. Table S1 shows the summary of the estimated average traveling speed and cost-weighted factors for potential terrain conditions that might be observed in our study area. While the cost-weighted factor represents the impedance which is inversely proportional to the average travel speed of each terrain type, a relative 'time-cost' surface or 'equivalent distance' surface can be produced for further modeling simulation.

**Table S1. Summary of the estimated average traveling speed and cost-weighted factor for potential terrain conditions in western Kenya**

| **Terrain Condition** | **Average Traveling Speed (km/hr.)** | **Cost-Weighted (Impedance) Factors** |
| --- | --- | --- |
| Primary Road (Bound Surface) (motorized transport) | 50 | 1 |
| Secondary Road (Loose Surface)  (motorized transport) | 30 | 1.667 |
| Dry Weather Road (earth surface)  (motorized transport) | 20 | 2.5 |
| Main Track  (motorized transport) | 10 | 5 |
| Footpath (by foot) | 5 | 10 |
| Off Road/path (by foot) | 3 | 16.667 |
| Forest (by foot) | 2.5 | 20 |
| Swamp (by foot) | 1 | 50 |
| River (inaccessible) | 0 | -1 |
