# Supplementary material for: Discovering the cost of care: consumer, provider, and retailer surveys shed light on the determinants of malaria health-seeking behaviours
Source: Malar J. 2016 Mar 22;15:179. doi: 10.1186/s12936-016-1232-7 (PMC4802645; doi:10.1186/s12936-016-1232-7)
Supplement: Supplementary file 2 — 10.1186/s12936-016-1232-7 Comparison between values measured by ELISA and HPLC in the commercial artemisinin-based drugs. The labelled value of active ingredients (a.i.) was all 2.0. [file 12936_2016_1232_MOESM2_ESM.docx]

**Table S2.** Comparison between values measured by ELISA and HPLC in the commercial artemisinin-based drugs. The labeled value of active ingredients (a.i.) was all 2.0

| Drug names | Lot No. | Site obtained | Measured content ^a^ (mg/mL) | |
| --- | --- | --- | --- | --- |
|  |  |  | ELISA | HPLC |
| Artefan 20/120 | P0251C | Kakamega, Kenya | 2.16 ± 0.03 | 2.33 ± 0.18 |
|  | BNP0501D | Emuhaya, Kenya | 2.38 ± 0.11 | 2.21 ± 0.01 |
|  | BNP0031D | Emuhaya, Kenya | 2.21 ± 0.23 | 2.22 ± 0.01 |
| CO-FALCINUM | B/NK 01885 | Vihiga, Kenya | 2.23 ± 0.21 | 2.17 ± 0.04 |
|  | B/NK 0C32 | Vihiga, Kenya | 2.16 ± 0.15 | 2.21 ± 0.01 |
|  | B/NK 01646 | Vihiga, Kenya | 2.38 ± 0.11 | 2.22 ± 0.12 |
|  | N/A b | Vihiga, Kenya | 2.39 ± 0.33 | 2.11 ± 0.02 |

|  | B/NK 10489 | Vihiga, Kenya | 2.22 ± 0.10 | 2.28 ± 0.03 |
| --- | --- | --- | --- | --- |
